# High levels of histone H3 K27 acetylation and tri-methylation are associated with shorter survival in oral squamous cell carcinoma patients

**DOI:** 10.37796/2211-8039.1391

**Published:** 2023-03-01

**Authors:** Akram Shahhosseini, Ekaterina Bourova-Flin, Samira Derakhshan, Pouyan Aminishakib, Afsaneh Goudarzi

**Affiliations:** a Department of Clinical Biochemistry, School of Medicine, Shahid Beheshti University of Medical Sciences, Tehran, Iran; b CNRS UMR 5309, INSERM U1209, University Grenoble-Alpes, Institute for Advanced Biosciences, La Tronche, France; c Oral and Maxillofacial Pathology Department, School of Dentistry, Tehran University of Medical Sciences, Tehran, Iran

**Keywords:** Histone modification, OSCC, Survival, Clinical significance

## Abstract

**Background:**

Acetylation and trimethylation of histone H3 lysine 27 (H3K27ac and H3K27me3) generally activate and repress transcription, respectively. Concurrent activation of H3K27ac and H3K27me3 has been reported to correlate with poor prognosis in hepatocellular carcinoma. A high level of H3K27me3 has been shown to be associated with advanced oral squamous cell carcinoma (OSCC) tumour stage, but prognostic impact of H3K27ac level alone/or in combination with H3K27me3 in OSCC patients had not yet been reported.

**Material and methods:**

In this study, immunohistochemistry using specific antibodies against H3K27ac and H3K27me3 was performed on a series of 72 OSCC samples to investigate the association between H3K27ac and H3K27me3 levels and OSCC patient’s survival. For each mark, the proportion of labelled cells (percentage) and the intensity of the labelling were measured, and a score of percentage x intensity was calculated.

**Results:**

A high percentage of H3K27me3 positive cells was significantly associated with survival in univariate and multivariate analyses (logrank p-value < 0.05). Patients with high total scores of H3K27ac and H3K27me3 labelling also showed significantly shorter survival probabilities (logrank p-value < 0.05) compared to patients with low total scores of labelling for these histone posttranslational modifications (PTMs).

**Conclusion:**

Our findings suggest that the detection of both H3K27ac and H3k27me3 could help evaluating prognosis in OSCC patients.

## 1. Introduction

Among all cancer types, 2%–4% is oral cancer which can occur in oral cavity, pharyngeal regions and salivary glands [1]. Oral squamous cell carcinoma (OSCC) accounts for the major subcategory of head and neck squamous cell carcinoma (HNSCC) and oral neoplasms [1]. The 5-year survival rate for OSCC patients is 40–50% and OSCC diagnosis often occurs in advanced stages [1,2]. Genetic changes and epigenetic alterations contribute to the malignant transformation of normal cells. Epigenetic mechanisms, including histone post-translational modifications (PTMs), DNA methylation and non-coding RNAs, are responsible for controlling gene expression patterns, which are essential for defining and maintaining cell identity. During cancer development and progression, a deregulation of the above-mentioned mechanisms resulting in altered expression of genes has been considered as a hallmark of cancer [3–6]. Acetylation and trimethylation of lysine 27 on histone H3 (H3K27ac and H3K27me3 respectively) are considered as two epigenetic marks with opposite effects, which respectively enhance and silence gene expression. H3K27ac is enriched at active enhancers and promoters whereas repressed enhancers are marked with H3K27me3 [7,8]. EZH2 is responsible for methylation at H3K27 and has been shown to be over-represented in many cancer types including head and neck, prostate and breast cancers [8–10]. Taking into account the importance of epigenetics in sustaining the normal functions of cells, the study of cancer epigenetics is a very promising emerging field. Different types of diseases including cancers have been reported to be associated with histone PTMs alterations [11,12]. Therefore, the detection of alterations in histone PTMs could be potentially used for more effective cancer diagnosis, prognosis and treatment. As opposed to genetic alterations, epigenetic changes are reversible and could potentially be targeted by epigenetic drugs aiming at restoring the original state of the transformed cell [13]. In the present study, we aimed to investigate the association between the survival of OSCC patients and H3K27ac and H3K27me3 levels, individually and in combination. For this purpose, specific antibodies against H3K27ac and H3K27me3 were used for the immunostaining of 72 OSCC samples.

## 2. Materials and methods

### 2.1. Subjects

Under ethical approval of Shahid Beheshti Medical University (IR.SBMU.MSP.REC.1397.572), 72 paraffin-embedded OSCC tissues were obtained from the archives of Oral and Maxillofacial Department, Tehran Medical University. Overall survival was available for all the patients. The follow-up data ranged from 1 to 80 months. Additionally, 2 fresh OSCC tissues were collected from Shariati and Bahman hospitals to investigate the specificity of histone PTMs antibodies. Tumour grades were scored as follows: grade I was defined as well-differentiated, grade II as moderately differentiated and grade III as poorly differentiated according to the WHO criteria. Clinical information such as age, gender, tumour location was collected from a review of medical records. The AJCC guidelines was applied for tumour staging.

### 2.2. Western blotting

Two Fresh frozen OSCC samples were lysed and homogenized in RIPA lysis buffer supplemented with protease inhibitor, PMSF and orthovanadate. Next, the cell extracts were subjected to sonication, which was followed by incubation on ice for 20 min and centrifuged at 12000rpm at 4°C for 20 min. Then, the supernatant was harvested and stored at −20°C until use. For the western-blotting, the protein extracts were loaded on 15% SDS-PAGE and separated proteins were transferred to nitrocellulose membrane, blocked with 3% BSA in TBST and incubated with the same antibodies as immunohistochemistry overnight at 4°C: H3K27me3 antibody (Cell signaling #9733, 1/1000), H3K27ac antibody (Abcam ab4729, 1/1000) and H3 antibody (CAT#PTM-1001, PTMbiolabs), the antibodies against H3K27ac and H3K27me3 are commercial, controlled and widely-used antibodies, and their specificity is well established [14–18]. After 16h incubation with the primary antibody, the membrane was washed 3 times with TBST and probed with secondary antibody for 1h at RT. Next, the membrane was washed again 3 times with TBST and the signal was visualized by ECL kit (GE Healthcare) on the X-ray film.

### 2.3. Immunohistochemical staining

Four-micrometer-thick sections from OSCC paraffin-embedded tissues were cut using a microtome and the sections were deposited on slides. Next, the slides were incubated at 60°C for 20min, deparaffinized in xylen and rehydrated in serial dilutions of ethanol (ethanol 100%, 96% and 70% for 2 min) and H2O which followed by incubation in PBS 3% hydrogen peroxide for 10min. The slides were then immersed in citrate buffer (pH = 6) and antigen retrieval was performed using microwave at 90°C for 15min. The sections were thereafter incubated with the primary antibodies, anti-H3K27ac (Abcam ab4729,1/200) and antiH3K27me3 (Cell signaling #9733, 1/200), for 1h at RT (For negative control PBS (phosphate buffered saline) was added instead of the primary antibody) which followed by three washes with PBS and then incubated with 100 ul of primary antibody amplifier master for 20min, after washing with PBS slides were incubated with 100 ul of secondary antibody for 30min, then washed 3 times in PBS. 1 drop of DAB chromogen concentrate was added to 1 drop of DAB substrate buffer (1:1) and 100 ul of it was used to develop the signal. After developing signal by DAB chromogen, the slides were dehydrated with serial dilutions of alcohol and mounted.

### 2.4. Evaluation of immunohistochemical staining

**Fig. 1:**
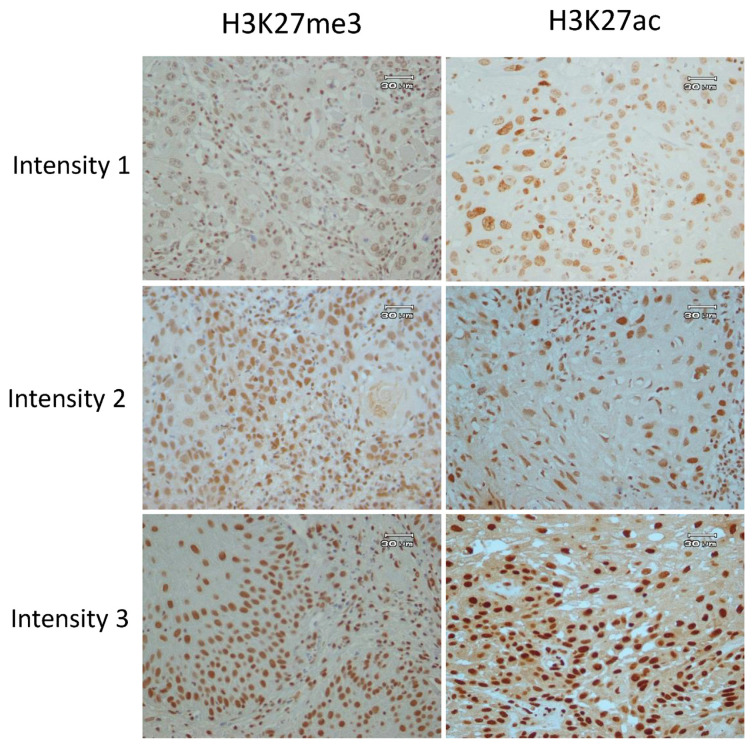
Images of OSCC tissue samples immunostained with H3K27ac and H3K27me3 illustrating the intensity scoring from 0 to 3 The scores of signal intensities were assigned for both of H3K27ac and H3K27me3 as: negative (0), weak (1), moderate (2) and strong (3) using a light microscope. Original magnification, 400X; Scale bar = 30 μm.

The stained slides were evaluated by two pathologists who were blinded to the clinicopathological information of patients. For both H3K27ac and H3K27me3, the percentage of positively stained tumour cells (“per”) and the scores of signal intensities (“in”: defined as negative (0), weak (1), moderate (2) and strong (3), illustrated in Fig. 1 were evaluated in 5 high power fields at 400X magnification using a light microscope. The total score (“perXin”) was calculated by multiplying the percentage of brown stained cells by the intensity score of the signal. The categories of high and low levels of H3K27me3 and H3K27ac were defined according to cut-off values of percentage and total scores as shown in Table 1.

### 2.5. Statistical analysis

The statistical analysis was performed using “scipy” package of Python software. The association between H3K27me3 and H3K27ac with clinicopathological features were investigated using Cox model and logrank test. Kaplan–Meier survival curves and the statistical tests of survival analysis were performed using “lifelines” package of Python. In all the analyses, a (p-value < 0.05) was considered statistically significant.

## 3. Results

### 3.1. The correlation of H3K27me3 and H3K27ac with clinical and pathological characteristics of patients

A total of 41 male and 24 female patients with a median age of 59.36 years (ranging from 36 to 80 years) were included in this study. The clinical and pathological characteristics of the patients are summarized in Table 2. Based on grading, 17 cases were grade I, 35 were grade II, 20 were grade III. The anatomical locations of OSCC included the oral cavity (5.55%), tongue (41.67%), lip/buccal mucosa (5.55%), gingiva/mandibular mucosa (12.5%), mouth floor (2.78%), maxilla (9.72%), mandibule (13.89%), palate (2.78%), and mandibular vestibule (5.55%). Among the patients for whom TNM staging was available (total n = 24), 16 cases (66.67%) had stage I, 3 cases (12.5%) had stage II and 5 (20.83%) had stage III. The correlation between the clinical and pathological features of the patients and tumours including age, gender, tumour site, grade and tumour size was investigated with different parameters of H3K27ac and H3K27me3, using the Pearson’s correlation coefficient and associated p-values for numerical variables (age, tumour size), and the analysis of variance (ANOVA statistical test) for other categorical variables. The results are shown in Table S1 and also illustrated in Fig. S3 for selected parameters with statistically significant correlations. We found that H3K27me3_per (proportion of cells labelled with H3K27me3) and combined H3K27_per (proportion of cells labelled with H3K27me3 or H3K27ac) were significantly anti-correlated with tumour size (p-value < 0.05). The Pearson’s correlation coefficient obtained for these values was equal to −0.25 and to −0.3, respectively, corresponding to a relatively mild correlation. For the patients with perineural invasion, we found that the corresponding acetylation percentage H3K27ac_per was statistically lower than for those without perineural invasion (Fig. S3C). Interestingly, both acetylation and methylation total scores (perXin) showed different values depending on the tumour site (Fig. S3A). For instance, we observed that the methylation total score H3K27me3_perXin had lower values in the gingiva/gum/mandibular mucosa and in the oral cavity as compared to the floor of the mouth or to the palate. For the acetylation total score H3K27ac_perXin, we found low values in the lip buccal mucosa and the oral cavity in comparison to the other tumour sites. Then, we compared the distributions of methylation and acetylation marks in each tumour site with ANOVA statistical test. We found that for three tumour sites (gingiva/ gum/mandibular mucosa, maxilla and mandibular vestibule) the acetylation scores of H3K27 were significantly higher than the methylation ones (Table S2 and Fig. S5). In addition, a survival analysis showed that overall survival prognosis of OSCC patients was significantly associated with tumour site in our dataset (logrank p-value = 0.012, Fig. S6A). This result is consistent with the previous studies [19,20], where a significant association between overall survival and the location of disease was discovered in large cohorts in Taiwan and in the United States.

### 3.2. H3K27me3 level is associated with shorter survival

**Fig. 2:**
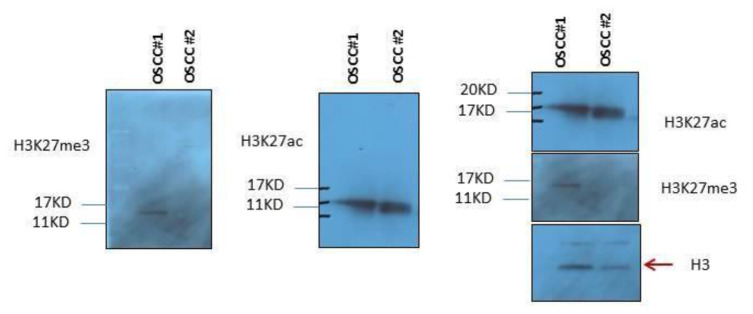
Western blot detection of H3K27ac and H3K27me3 in tumour samples The total protein extracts were prepared from two OSCC tissues (OSCC#1 and OSCC#2) and western blot was performed using H3K27ac and H3K27me3 antibodies. The bands detected by H3K27ac and H3K27me3 antibodies demonstrate the correct and expected size and reveal the specificity of antibodies used. Histone H3 was used as a loading control.

To ensure that H3K27ac and H3K27me3 antibodies recognize acetylation and methylation solely on lysine 27 of histone H3, the specificity of antibodies was confirmed by immunoblotting using OSCC protein extracts, with H3K27ac and H3K27me3 antibodies (Fig. 2). These antibodies were then used for immunohistochemistry (IHC) on the tumour sections from the cohort of OSCC patients, and values corresponding to the percentage of positive cells, the labelling intensity and the score of percentage x intensity were calculated for each tumour and each antibody, as detailed in the methods section (Fig. 1 and Table 1).

We then looked for an association between the levels of H3K27ac and H3K27me3 labelling and survival using two approaches. In the first approach, we explored the association with survival of H3K27me3 and H3K27ac levels using univariate Cox proportional hazard model. This model performs a regression for each histone PTM mark with respect to the survival follow-up data to estimate the impact of the corresponding histone PTM level on patient’s survival probability, evaluated by a p-value and a hazard ratio. We considered that the association with survival was statistically significant if the obtained p-value was smaller than 0.05. The Cox proportional hazard model was computed separately for each histone PTM mark (methylation and acetylation), and for the percentage (proportion of labelled cells) and total score (labelling intensity x percentage of labelled cells). The results, including p-values, hazard ratios and confidence intervals for hazard ratios, calculated for each explanatory variable, are presented in Table 3. We found that only the percentage of H3K27me3 labelled cells (H3K27me3_per) was significantly associated with survival (p-value = 0.034, see Table 3). This result means that a higher H3K27me3_per level is associated with shorter survival probability. The other parameters were not directly associated with survival when considering their measured levels.

**Fig. 3:**
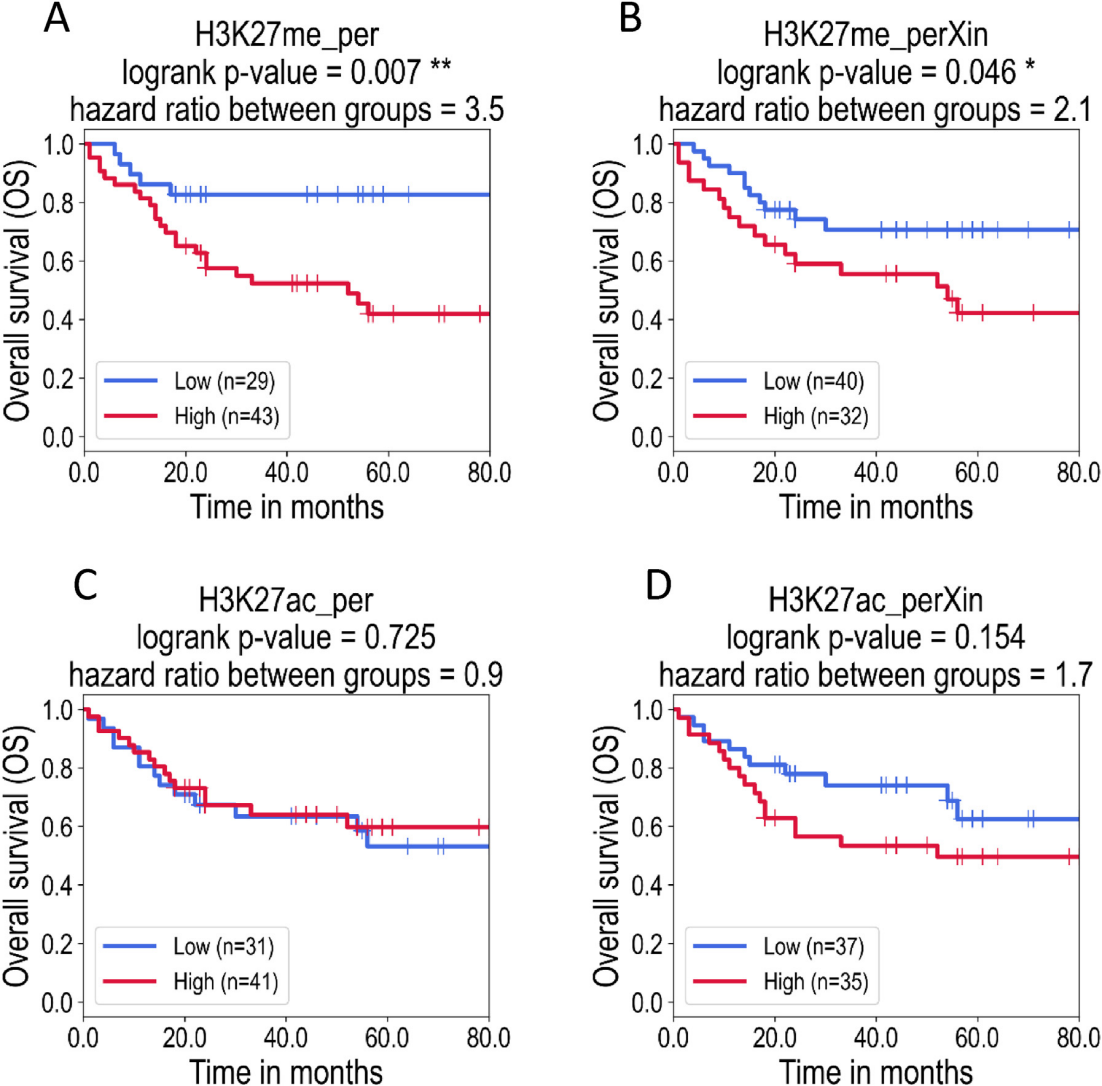
Kaplan–Meier survival curves of OSCC patients according to the percentage of labelled cells (per) (A and C) and percentage x intensity (perXin) scores (B and D) of H3K27me3 (A, B) or H3K27ac (C, D) labelling (A) The patients were stratified in two groups of high and low values according to the percentage of cells stained with H3K27me3 (H3K27me3_per). The logrank survival analysis showed significant association between the percentage of H3K27me3 labelled cells with overall survival of OSCC patients (p-value < 0.05). (B) Similarly, the logrank test of patients with high and low total scores of H3K27me3 (H3K27me3_perXin) showed a statistically significant association with survival. (C and D) Although a high total intensity x percentage score of H3K27ac (H3K27ac_perXin) tends to be associated with poor prognosis, neither H3K27ac_per nor H3K27ac_perXin were significantly associated with survival. In all the plots, the star symbol marks statistically significant p-values: *: p-value < 0.05, p-value < 0.001.

In the second approach, a threshold was defined for each of the measured levels of histone PTM marks, the values were binarized into “low” (0) or “high” (1) levels for each parameter, and the patients were grouped accordingly. The position of the threshold to separate “low” and “high” groups was selected in order to obtain two sample groups of similar sizes (Fig. S1). We then performed a logrank test to find if the survival probability was significantly different between the patients of the two groups. The association with survival was considered significant if the logrank p-value was less than 0.05. We found that the percentage of cells labelled with H3K27me3 (H3K27me3_per) and the total score of H3K27me3 (intensity x percentage of labelled cells: H3K27me3_perXin) were significantly associated with shorter survival (Fig. 3A and B). Hence, patients whose tumours showed a high percentage of positively H3K27me3 stained cells (high values of H3K27me3_per) and high H3K27me3_perXin scores were both significantly associated with shorter survival compared to patients with low H3K27me3 labelling. H3K27ac labelling was not found to be significantly associated with overall survival (Fig. 3C and D).

The results of both approaches are consistent and confirm that H3K27me3 can be used as a predictive biomarker for survival, by directly considering the proportion of H3K27me3 labelled cells or by creating groups of respectively low and high levels of H3K27me3 labelled cells (H3K27me3_per), or groups of low and high H3K27me3 scores (H3K27me3_perXin).

Regarding the total acetylation score H3K27ac_-perXin, although the trend of the survival curve is consistent with the conclusions, the sample size was not sufficient to reach statistical significance (p-value = 0.154).

### 3.3. Combination of H3K27ac and H3K27me3 is associated with shorted survival in OSCC patients

**Fig. 4:**
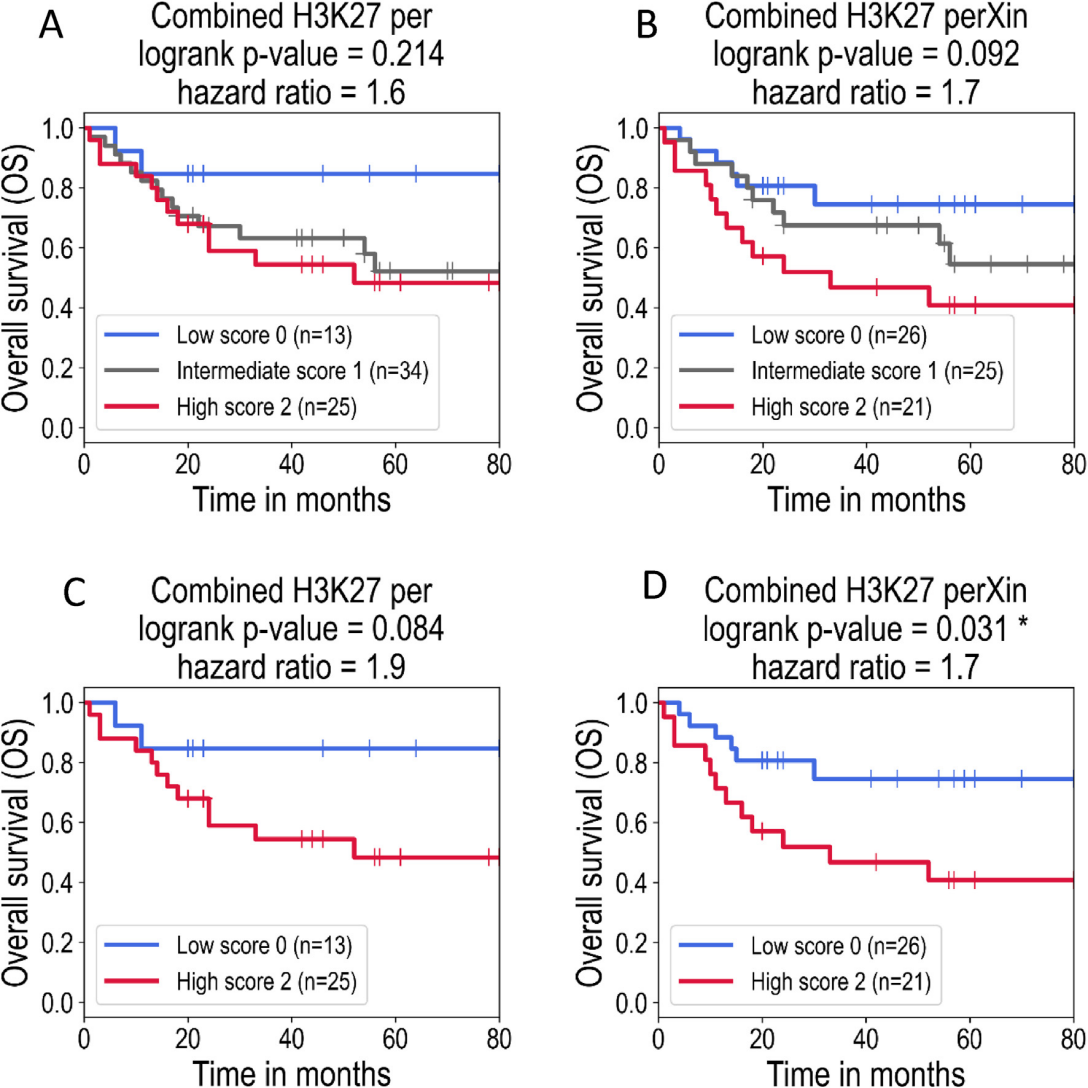
Kaplan–Meier survival curves according to the combined scores of H3K27me3 and H3K27ac in OSCC patients. In these plots, the patients were stratified in three groups according to the number of marks measured above the threshold as follows: low score 0 = both H3K27ac and H3K27me3 marks are below the threshold, intermediate score 1 = only one of the two marks is above the threshold and high score 2 = both marks are above the threshold. (A) Kaplan–Meier survival curves and the results of the logrank test for the combination of H3K27ac_per and H3K27me3_per (combined H3K27_per). (B) Same for the combination of H3K27ac_perXin and H3K27me3_perXin (combined H3K27_perXin). (C) Same as Fig. 4A, showing only two groups out of three: low score 0 and high score 2 (the group of the intermediate score 1 is excluded). (D) Same as Fig. 4B, showing only two groups out of three: low score 0 and high score 2 (the group of the intermediate score 1 is excluded). The symbol * indicates statistically significant p-value < 0.05 of the logrank test.

As shown in (Fig. S2), H3K27ac and H3K27me3 levels are not correlated (Pearson’s correlation coefficient is equal to 0.07 and 0.22 respectively, p-values are not significant) suggesting that H3K27 acetylation and methylation values can be considered as independent variables. It was therefore interesting to explore if not only the individual histone PTM marks but also a combination of both H3K27ac and H3K27me3 marks was associated with survival. For each parameter, we separated patients in three groups according to the number of marks measured above the threshold as follows: 0 = both H3K27ac and H3K27me3 values are below the threshold, 1 = only one of the two values is above the threshold and 2 = both values are above the threshold. We then compared the survival probabilities between the groups using the logrank test. We found that the combined score perXin (proportion of labelled cells x labelling intensity) of both marks was significantly associated with survival. Indeed, patients with a high score (=2, as defined above) of combined H3K27me3 and H3K27ac labelling showed a significantly shorter survival compared to patients with low (=0, as defined above) scores (p-value = 0.031, Fig. 4D). The result obtained using the combined value of percentages, although not formally significant (p-value = 0.084), showed the same tendency (Fig.4A and C). In addition, we calculated survival probabilities between all possible groups of low and/or high status of acetylation and methylation marks (see Fig. S4).

The association with overall survival was found to be significant for the combined percentage of H3K27 (p-value = 0.046) and not significant for the combined intensity (p-value = 0.215) or perXin score (p-value = 0.174). While considering two extreme groups (low acetylation and methylation status versus high acetylation and methylation status), excluding intermediate combinations, we found significant association with survival for both the combined H3K27 intensity and perXin the scores (Fig.S4E-F). The trend of the percentage score is consistent with our conclusions but the number of patients were too low to reach statistical significance (p-value = 0.084, Fig. S4D).

Interestingly, the impact of methylation status on survival probability for the combined H3K27 percentage is more important than acetylation, while the situation seems to be opposite for the combined H3K27 intensity. These results suggest to use preferentially the perXin score, considering the impact of both percentage and intensity of H3K27 on survival probability, as well as the combination of acetylation and methylation.

As the tumour location represents a risk factor that can potentially influence the prognosis in OSCC, it was interesting to investigate the impact of both combined histone PTM marks and tumour sites on survival probability. Unfortunately, the sample size in our cohort was too small to perform a survival analysis in individual tumour subsites. Therefore, we created two pooled groups of anatomic sites with 36 samples in each group. The group “Tongue-FOM” contains tumour samples of tongue, floor of the mouth (FOM) and oral cavity. The other anatomic sites were included in the group “Other sites”. The overall survival for OSCC patients in the group “Tongue-FOM” was found significantly longer than in other tumour sites (logrank p-value = 0.004, Fig. S6B). We computed the distribution of OSCC samples in the pooled groups according to the combined H3K27 methylation and acetylation scores (Fig. S6C). We found a quite ubiquitous distribution of samples within each group, suggesting that the combined H3K27 score brings independent and complementary information compared to tumour site. This conclusion is confirmed by a subsequent survival analysis in each group of tumour sites. Indeed, the patients with a low H3K27 methylation and acetylation score have a tendency for a better survival prognosis than the patients with a high H3K27 methylation and acetylation score, both in the group “Tongue-FOM” and in “Other sites” (Fig. S6C). However, the logrank p-values obtained in subgroups of tumour sites were not formally significant (p-value < 0.2) due to small sample sizes.

## 4. Discussion

The lysine acetylation and methylation of histone proteins are specific transcriptional regulators [21]. Trimethylation at lysine 27 of histone H3 is a marker of silenced genes whereas acetylation at lysine 27 of histone H3 is associated with active transcription [22]. Altered patterns of histone modifications result in deregulated gene expression and have been reported to contribute to cancer development and progression [23]. The high mortality and poor prognosis of OSCC patients require novel therapeutic strategies to fight this cancer. In the current study, the results using IHC staining show that histone H3K27ac and H3K27me3 are independent and complementary marks which enable us to investigate the association of these histone PTM marks with the survival of OSCC patients individually and in combination. A positive association between the percentage of stained H3K27me3 and OSCC survival was consistent with the result reported by [24]. Indeed, the percentage of cells labelled by H3K27me3 (H3K27me3_per) as well as high-total scoring of H3K27me3 (H3K27me3_-perXin) tend to be associated with shorter survival. Although many histone modifications have been studied in OSCC patients [24,25], the association of H3K27ac, a mark of active transcription, with survival outcome of OSCC patients has remained unclear. Here for the first time, by combining the scores of H3K27ac and H3K27me3, we show that concurrent high levels of H3K27ac and H3K27me3 show a tendency to be associated with OSCC patients’ poor prognosis. Therefore, the sum of total scores of H3K27ac and H3K27me3 could be used to stratify patients into two prognosis groups of “high” and “low” levels of acetylation and methylation, with a shorter survival for patients in the “high” group. Each histone inside the nucleosome is subject to a variety of histone PTMs, and the IHC gives us only a global evaluation of the amounts of each specific histone PTM. In order to understand through which mechanism high levels of H3K27ac and H3K27me3 could be associated with poor prognosis, one would need to perform transcriptomic and epigenomic analyses on OSCC and normal mucosa tissues. For instance, chromatin immunoprecipitation using anti-H3K27ac and anti-H3K27me3 from OSCC and from normal mucosa tissue could unravel which genes are enriched in these two histone PTMs and enable us to compare their expression levels between tumours and normal mucosa tissue. The expression levels of p300/CBP and EZH2 would also need to be evaluated, since these factors are respectively writers of H3K27ac and H3K27me3 [26]. The aberrant expression and mutation of p300/CBP and EZH2 has been reported in human cancers [27–31]. The inhibition of p300/CBP has been shown to impair the expression of oncogenic transcription factors [27]. Indeed, cellular transcription is governed by a finely tuned steadystate equilibrium between histone H3K27 acetylation and methylation [27]. A shift from this equilibrium towards either acetylation or methylation could favour an oncogenic gene expression program [27] and hence could explain why we found here that both H3K27 acetylation and methylation are indicative of poor prognosis.

Altogether, our findings demonstrate that elevated acetylation and methylation of H3K27 correlate with shorter OSCC patients’ survival. Considering the fact that deregulated H3K27 acetylation or methylation could impair the gene expression profiles of cells, the corresponding writers of these marks could be good candidates as therapeutic targets to reverse the effect of deregulated levels of histone modification [32,33].

## Figures and Tables

**Table 1:** Immunohistochemistry scoring of samples stained with anti-H3K27me3 and anti-H3K27ac.

	Score	High	Low
H3K27me3	Percentage	>89.5	≤89.5
	Total (perXin)	>189.5	≤189.5
H3K27ac	Percentage	>99.5	≤99.5
	Total (perXin)	>269.5	≤269.5

**Table 2:** Clinicopathological characteristics of OSCC patients (n = 72).

Characteristics	No.	%
Age (y)		
Mean	3.42	
Age (y)		
<55	27	40.29
>55	40	59.7
Sex		
Female	24	36.92
Male	41	63.07
Grade		
Grade I	17	23.61
Grade II	35	48.61
Grade III	20	27.77
Stage		
I	16	66.67
II	3	12.5
III	5	20.83
Death		
Yes	31	43
No	41	57
Vascular invasion		
Yes	3	5
No	60	95
Perineural invasion		
Yes	5	8
No	58	92
Tumor subsites		
Oral cavity	4	5.55
Tongue	30	41.67
Mouth floor	2	2.78
Lip/Buccal mucosa	4	5.55
Palate	2	2.78
Maxilla	7	9.72
Mandibule	10	13.89
Mandibular vestibule	4	5.55
Gingiva/mandibular	9	12.5
mucosa		

**Table 3:** Results of univariate Cox proportional hazard model obtained for histone PTM marks.

parameter	p-value	hazard ratio (HR)	HR CI 95%
H3K27me_per	0.034*	1.03	1.00-1.0
H3K27me_perXin	0.148	1.00	1.00-1.01
H3K27ac_per	0.175	0.99	0.97-1.01
H3K27ac_perXin	0.405	1.00	1.00-1.01

* indicates statistically significant p-value < 0.05

## Appendix

Fig. S1 Distributions, thresholds and sample sizes in groups for the main parameters of H3K27me3 and H3K27ac. Distributions of histone PTM measurements presented as Kernel Density Estimation (KDE) plots. The position of a threshold to separate “low” and “high” groups for each histone PTM mark is shown in grey dashed lines. The thresholds were selected to separate the mostly equally the samples in two groups (median values).

Fig. S2 Correlations between the main parameters of H3K27ac and H3K27me3. (A) Correlation between H3K27ac_per and H3K27me3_per. (B) Correlation between H3K27ac_perXin and H3K27me3_perXin. The Pearson’s correlation coefficient (correlation value) and the associated p-value are shown for each plot.

Fig. S3 Correlations between the main parameters of H3K27ac and H3K27me3 and patients’ clinical and pathological characteristics. (A) Distribution of the acetylation and methylation total score (perXin) in different tumour sites. (B) Correlations of H3K27me3_per and combined H3K27_per with tumour size. (C) Distribution of H3K27ac_per values in two groups of patients, with perineural invasion and without perineural invasion. The symbols *, ** and *** indicate statistically significant p-value < 0.05, p-value < 0.01 and p-value < 0.001, respectively.

Fig. S4 Kaplan–Meier survival curves according to the combined status of H3K27me3 and H3K27ac in OSCC patients. (A–C) Kaplan–Meier survival curves and the results of the logrank test in combined groups of low and/or high status of methylation and acetylation for percentage (A), intensity (B) and perXin score (C) of H3K27. (D–F) Same survival curves considering only two groups: one group with low status of both methylation and acetylation (green line) and another group with high status of both methylation and acetylation (purple line). In plot legends, “me” stands for methylation and “ac” for acetylation. The symbol * indicates statistically significant p-value < 0.05 of the logrank test.

Fig. S5 Boxplots of H3K27 methylation and acetylation marks in different anatomical tumour sites. (A–C) Percentage of H3K27 methylation and acetylation marks for three anatomical sites: gingiva / gun / man. mucosa (A), maxilla (B) and man. vestibule (C). (D–F) Same for H3K27 perXin score. In plot legends, “me” stands for methylation and “ac” for acetylation. Individual measurements are shown in green dots. The significance symbols of ANOVA p-values are the following: * for p-value < 0.05, ** for p-value < 0.01 and *** for p-value < 0.001.

Fig. S6 Combined H3K27 methylation and acetylation scores in different anatomical tumour sites. (A) Kaplan–Meier plots of overall survival corresponding to different anatomical sites of OSCC. (B) Kaplan–Meier plots of overall survival in two pooled groups of anatomical sites. The group “Tongue-FOM” contains tumour samples of tongue, floor of the mouth and oral cavity. The other anatomical sites are included in the group “Other sites”. (C) Distribution of samples in pooled anatomical sites according to combined H3K27 percentage (per) and perXin scores in OSCC patients. (D) Kaplan–Meier plots showing overall survival for different combined H3K27 percentage (per) and perXin scores within the groups of pooled anatomical sites. The significance symbols of p-values are the following: * for p-value < 0.05 and ** for p-value < 0.01.

**Table S1:** Correlations between the main parameters of H3K27ac and H3K27me3 and patients’ clinicopathologic characteristics. The table shows Pearson’s correlation coefficients and the associated p-values, calculated for numerical variables (age and tumour size), as well as the results of the ANOVA statistical test, calculated for categorical variables (including age groups, gender, grade, perineural invasion, site, TNM stage, tumour size, vascular invasion, alcohol and smoking). The symbols ^*^, ^**^ and ^***^ indicate statistically significant p-value < 0.05, p-value < 0.01 and p-value < 0.001, respectively. The statistically significant results (p-value < 0.05) are marked in red.

Parameters	Sample site	Groups	H3K27me3_per	H3K27me3_perXin	H3K27ac_per	H3K27ac_perXin	combined_H3K27_per	combined_H3K27_perXin
Age	n=67	-	Pearson’s corr. coef.=0.07 pval=5.9e-01	Pearson’s corr. coef.=−0.04 pval=7.6e-01	Pearson’s corr. coef.=0.01 pval=9.4e-01	Pearson’s corr. coef.=0.06 pval=6.3e-01	Pearson’s corr. coef.=0.06 pval=6.1e-01	Pearson’s corr. coef.=0.01 pval=9.6e-01
Age group	n=67	< 55 years (n=27),> 55 years (n=40)	ANOVA pval=8.7e-01	ANOVA pval=6.4e-01	ANOVA pval=4.5e-01	ANOVA pval=1.8e-01	ANOVA pval=8.8e-01	ANOVA pval=6.9e-01
gender	n=65	F (n=24), M (n=41)	ANOVA pval=3.7e-01	ANOVA pval=4.5e-01	ANOVA pval=8.0e-01	ANOVA pval=5.3e-01	ANOVA pval=5.4e-01	ANOVA pval=8.7e-01
Grade	n=72	G1 (n=17), G2 (n=35), G3 (n=20)	ANOVA pval=9.0e-01	ANOVA pval=3.7e-01	ANOVA pval=7.8e-02	ANOVA pval=2.0e-01	ANOVA pval=3.6e-01	ANOVA pval=2.9e-01
Perineural Invasion	n=63	No (n=58), Yes (n=5)	ANOVA pval=3.7e-01	ANOVA pval=8.1e-01	ANOVA pval=2.1e-02^*^	ANOVA pval=5.8e-01	ANOVA pval=8.0e-01	ANOVA pval=8.9e-01
Site	n=72	Floor of the mouth (n=2),Gingiva/gum/man. mucosa (n=9), Lip/ Buccal Mucosa (n=4), Man. Vestibule (n=4), Mandibule (n=10), Maxilla (n=7). Palate (n=2), oral cavity (n=4), tongue (n=30)	ANOVA pval=8.7e-02	ANOVA pval=6.8e-03^**^	ANOVA pval=9.0e-01	ANOVA pval=4.7e-02^*^	ANOVA pval=2.4e-01	ANOVA pval=8.5e-02
TNM stage	n=24	T1 (n=16),T2 (n=3), T3 (n=5)	ANOVA pval=7.4e-01	ANOVA pval=9.1e-01	ANOVA pval=1.6e-03^**^	ANOVA pval=4.9e-03^**^	ANOVA pval=1.1e-01	ANOVA pval=1.3e-01
Tumour size	n=72	-	Pearson’s corr. coef.=−0.25 pval=3.4e-02^*^	Pearson’s corr. coef.=0.12 pval=3.0e-01	Pearson’s corr. coef.=−0.19 pval=1.1e-01	Pearson’s corr. coef.=0.03 pval=8.1e-01	Pearson’s corr. coef.=−0.30pval=9.7e-03^**^	Pearson’s corr. coef. =−0.07 pval=5.6e-01
Vascular Invasion	n=63	No (n=60), Yes (n=3)	ANOVA pval=3.2e-01	ANOVA pval=5.9e-01	ANOVA pval=6.2e-02	ANOVA pval=8.0e-01	ANOVA pval=9.9e-01	ANOVA pval=6.0e-01
Alcohol	n=52	No (n=51), Yes (n=1)	ANOVA pval=0.6896	ANOVA pval=0.9503	ANOVA pval=0.4838	ANOVA pval=0.3563	ANOVA pval=0.945	ANOVA pval=0.5573
Smoking	n=52	No (n=45), Yes (n=7)	ANOVA pval=0.6374	ANOVA pval=0.842	ANOVA pval=0.0039^**^	ANOVA pval=0.2717	ANOVA pval=0.059	ANOVA pval=0.4339

**Table S2:** Correlations between the main parameters of H3K27ac and H3K27me3 and patients’ tumour site. The table shows the results of the ANOVA statistical test, calculated for different staining patterns of H3K27ac and H3K27me3 depending on tumour site. The symbols ^*^ and ^***^ indicate statistically significant p-value < 0.05 and p-value < 0.001, respectively. The statistically significant results (p-value < 0.05) are marked in red.

Site	Sample size	H3K27me_per vs. H3K27ac_per	H3K27me_perXin vs. H3K27ac_perXin
Floor of the mouth	2	ANOVA pval = 0.4726	ANOVA pval = 0.4226
Gingiva / gum / man. mucosa	9	ANOVA pval = 0.0158^*^	ANOVA pval = 0.0000 ^***^
Lip / Buccal Mucosa	4	ANOVA pval = 0.6202	ANOVA pval = 0.8905
Man. Vestibule	4	ANOVA pval = 0.0401 ^*^	ANOVA pval = 0.0001 ^***^
Mandibule	10	ANOVA pval = 0.4476	ANOVA pval = 0.5067
Maxilla	7	ANOVA pval = 0.0442 ^*^	ANOVA pval = 0.0430 ^*^
Oral cavity	4	ANOVA pval = 0.1955	ANOVA pval = 0.4916
Palate	2	ANOVA pval = 1.0000	ANOVA pval = 1.0000
Tongue	30	ANOVA pval = 0.1690	ANOVA pval = 0.4672
