# Supplementary figures and images for: High levels of histone H3 K27 acetylation and tri-methylation are associated with shorter survival in oral squamous cell carcinoma patients
Source: Biomedicine (Taipei). 2023 Mar 1;13(1):22–38. doi: 10.37796/2211-8039.1391 (PMC10166250; doi:10.37796/2211-8039.1391)

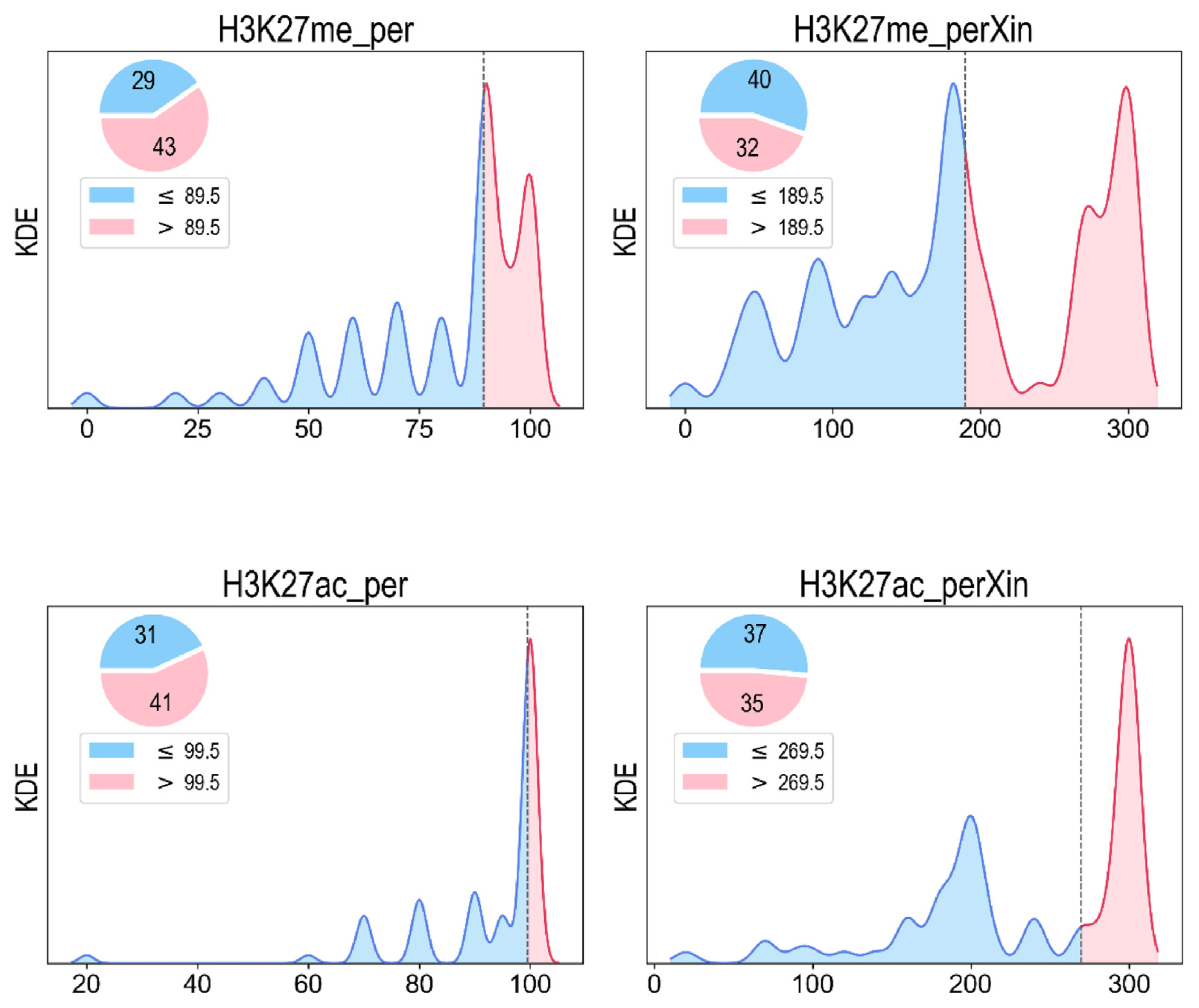

Supplement: Fig. S1 — Distributions, thresholds and sample sizes in groups for the main parameters of H3K27me3 and H3K27ac. Distributions of histone PTM measurements presented as Kernel Density Estimation (KDE) plots. The position of a threshold to separate “low” and “high” groups for each histone PTM mark is shown in grey dashed lines. The thresholds were selected to separate the mostly equally the samples in two groups (median values). [file bmed-13-01-022s1.tif]

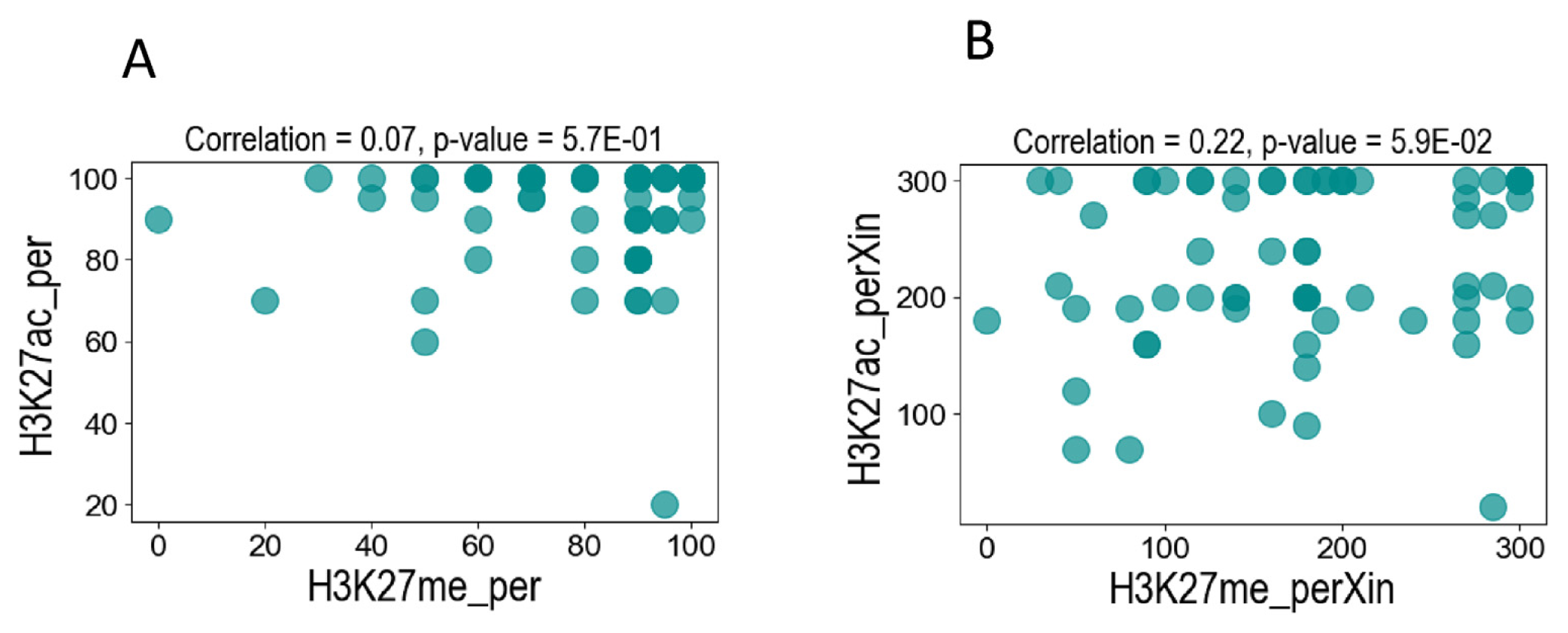

Supplement: Fig. S2 — Correlations between the main parameters of H3K27ac and H3K27me3. (A) Correlation between H3K27ac_per and H3K27me3_per. (B) Correlation between H3K27ac_perXin and H3K27me3_perXin. The Pearson’s correlation coefficient (correlation value) and the associated p-value are shown for each plot. [file bmed-13-01-022s2.tif]

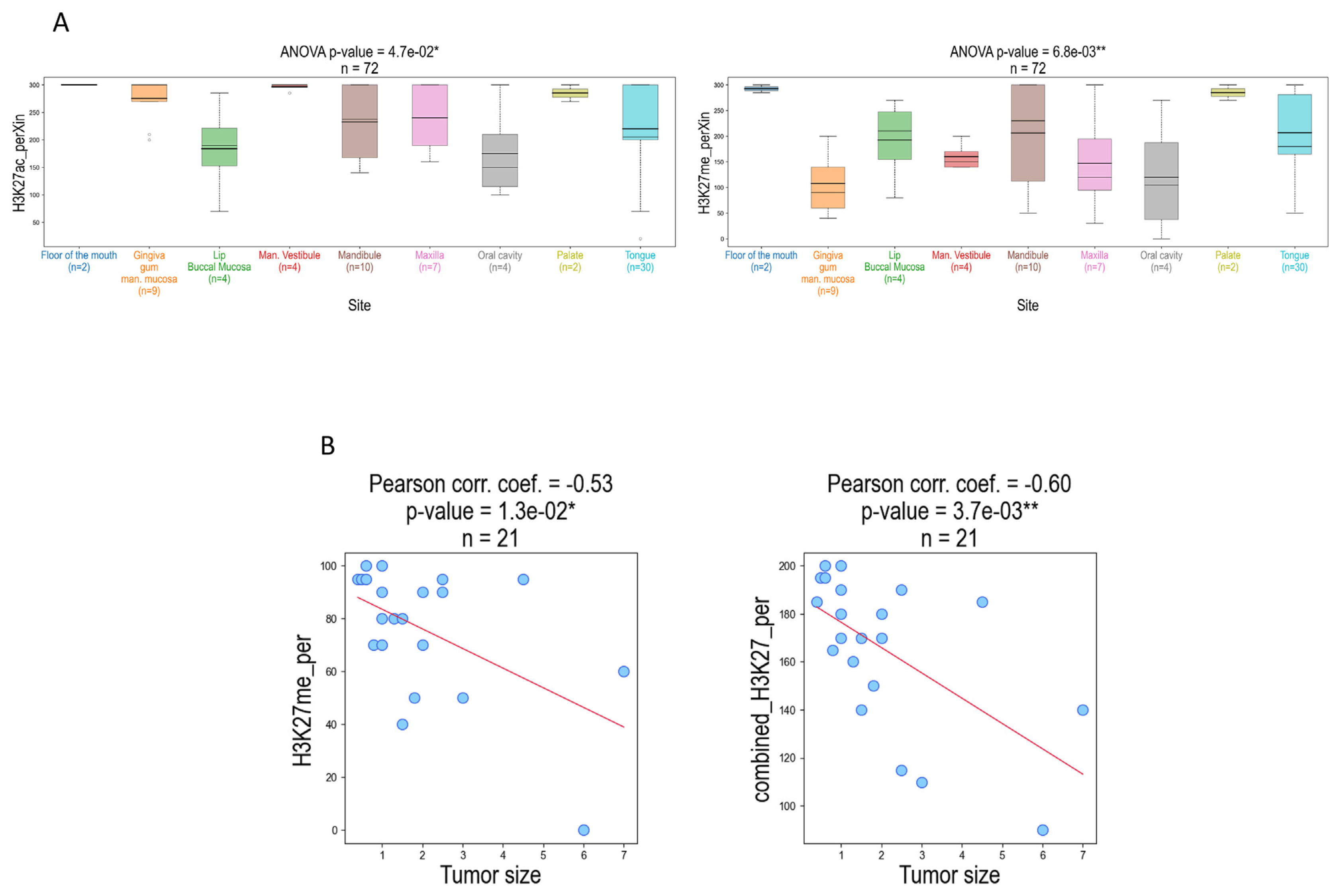

Supplement: Fig. S3 — Correlations between the main parameters of H3K27ac and H3K27me3 and patients’ clinical and pathological characteristics. (A) Distribution of the acetylation and methylation total score (perXin) in different tumour sites. (B) Correlations of H3K27me3_per and combined H3K27_per with tumour size. (C) Distribution of H3K27ac_per values in two groups of patients, with perineural invasion and without perineural invasion. The symbols *, ** and *** indicate statistically significant p-value < 0.05, p-value < 0.01 and p-value < 0.001, respectively. [file bmed-13-01-022s3.tif]

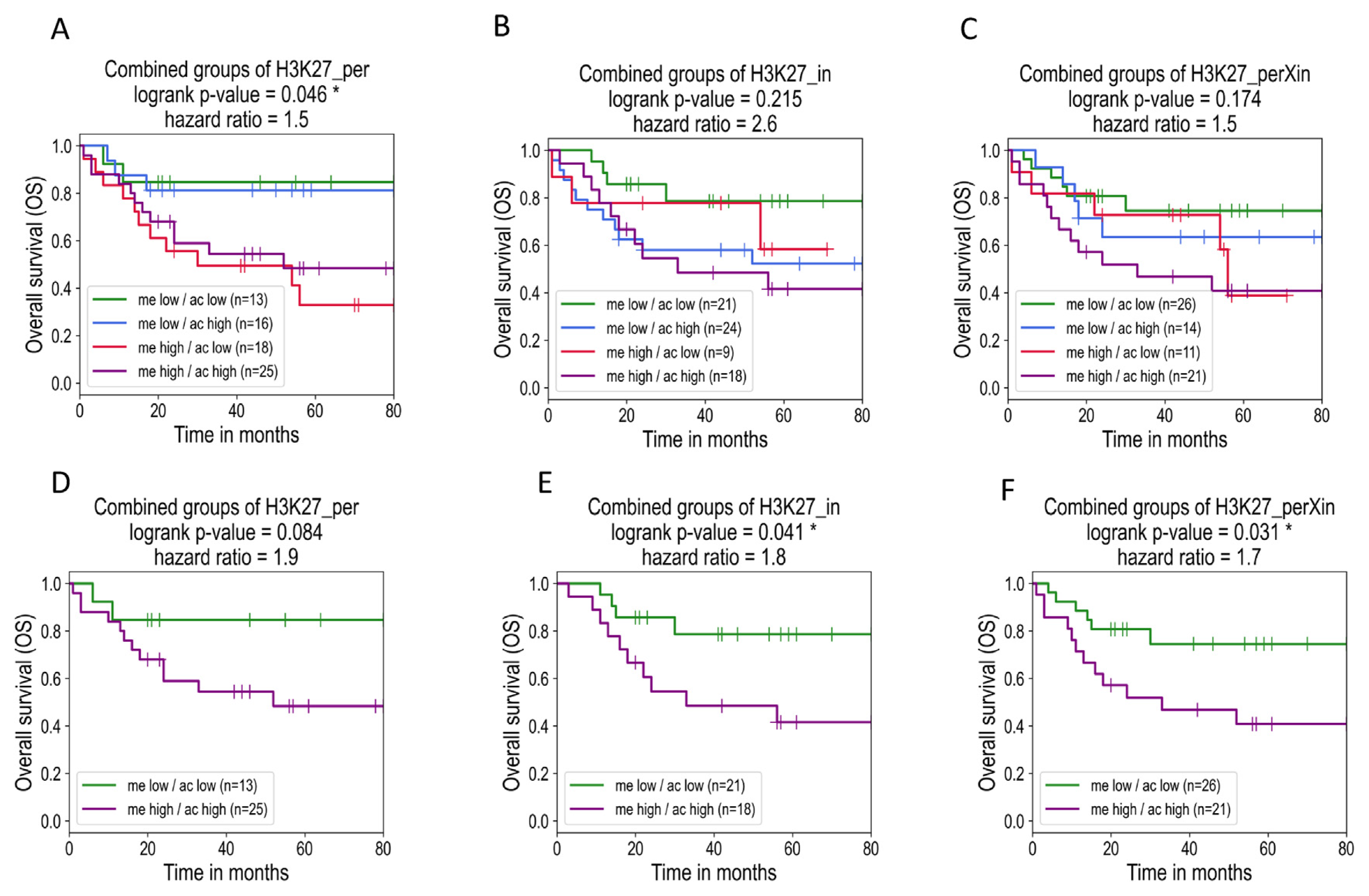

Supplement: Fig. S4 — Kaplan–Meier survival curves according to the combined status of H3K27me3 and H3K27ac in OSCC patients. (A–C) Kaplan–Meier survival curves and the results of the logrank test in combined groups of low and/or high status of methylation and acetylation for percentage (A), intensity (B) and perXin score (C) of H3K27. (D–F) Same survival curves considering only two groups: one group with low status of both methylation and acetylation (green line) and another group with high status of both methylation and acetylation (purple line). In plot legends, “me” stands for methylation and “ac” for acetylation. The symbol * indicates statistically significant p-value < 0.05 of the logrank test. [file bmed-13-01-022s4.tif]

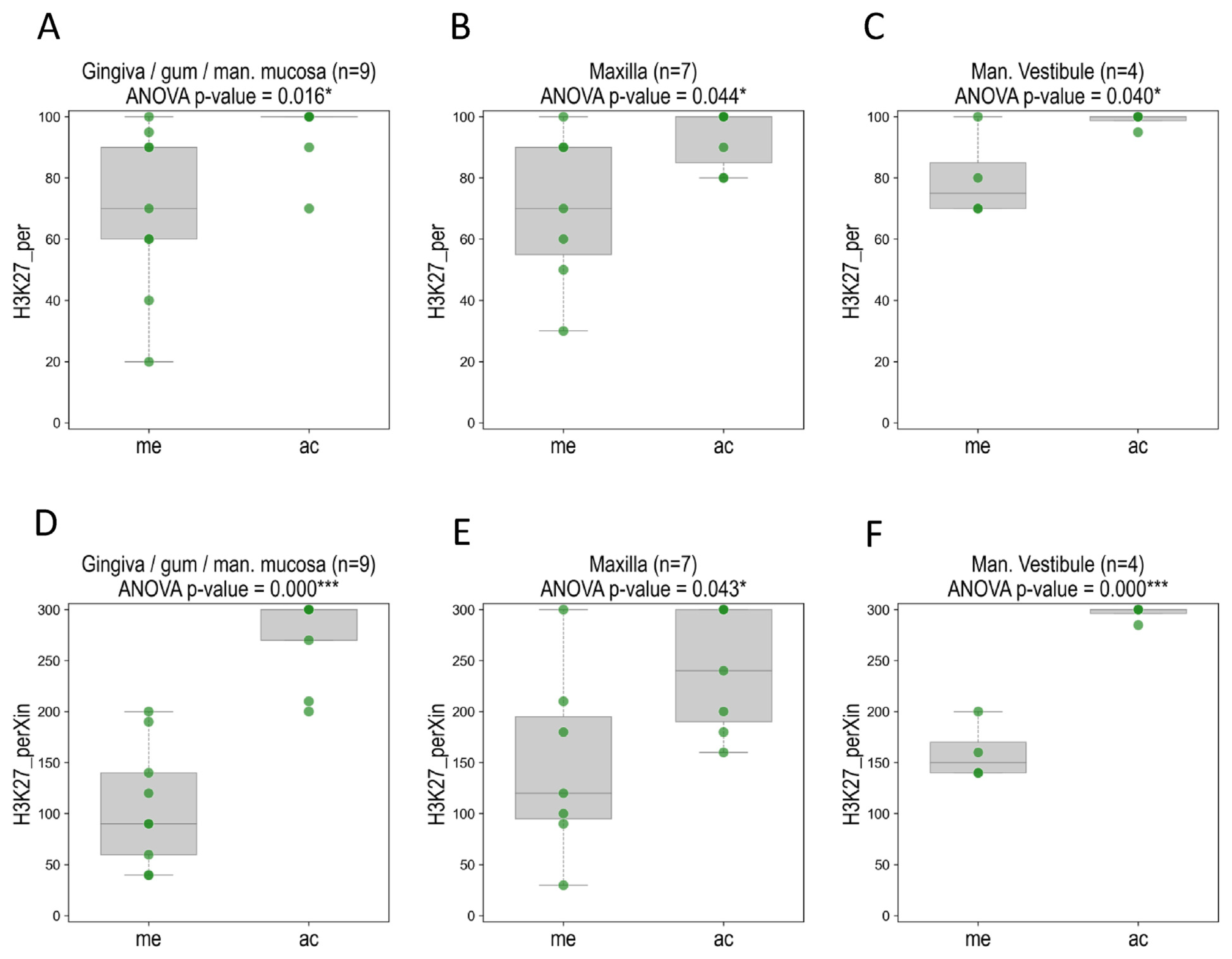

Supplement: Fig. S5 — Boxplots of H3K27 methylation and acetylation marks in different anatomical tumour sites. (A–C) Percentage of H3K27 methylation and acetylation marks for three anatomical sites: gingiva / gun / man. mucosa (A), maxilla (B) and man. vestibule (C). (D–F) Same for H3K27 perXin score. In plot legends, “me” stands for methylation and “ac” for acetylation. Individual measurements are shown in green dots. The significance symbols of ANOVA p-values are the following: * for p-value < 0.05, ** for p-value < 0.01 and *** for p-value < 0.001. [file bmed-13-01-022s5.tif]

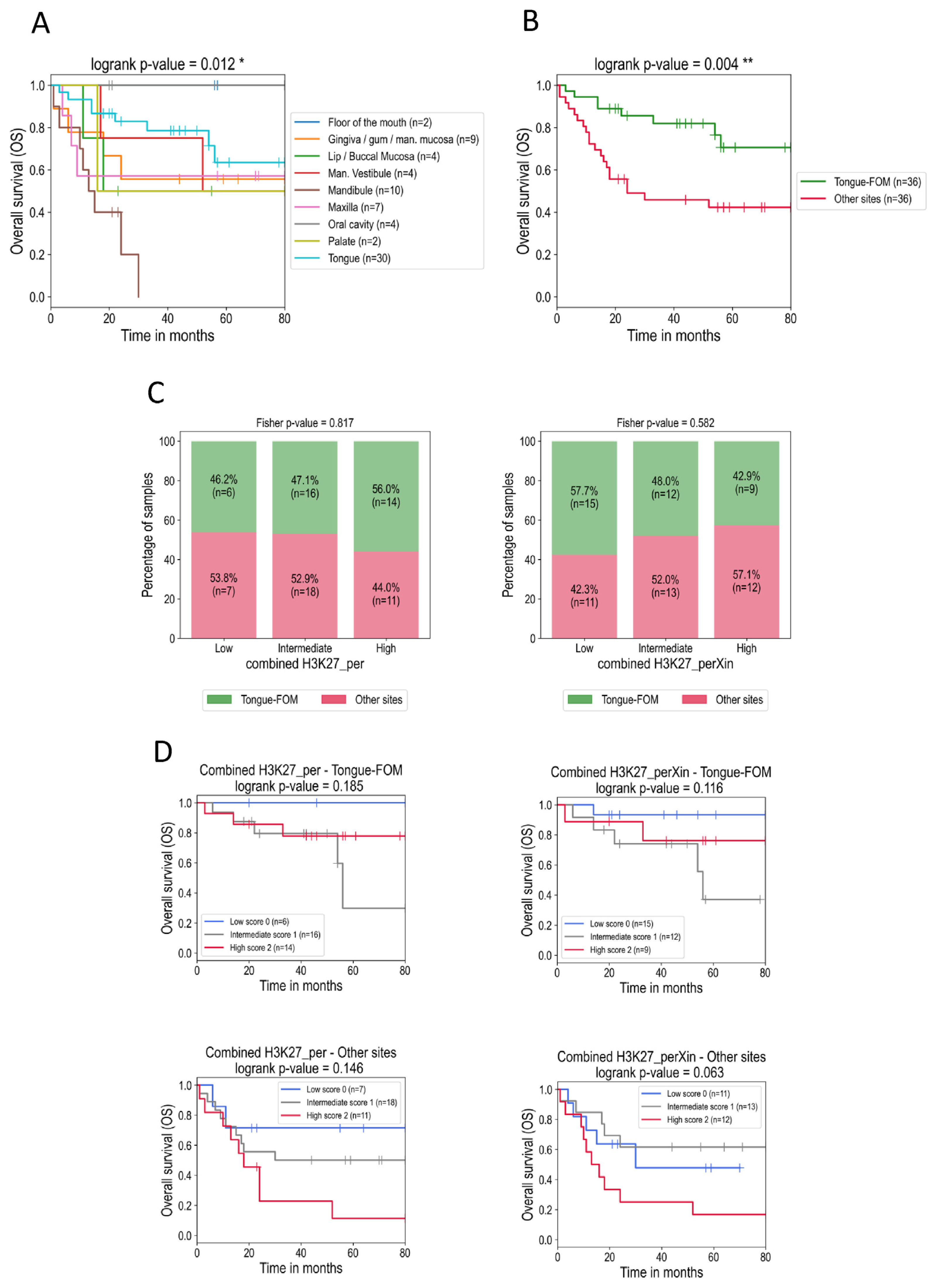

Supplement: Fig. S6 — Combined H3K27 methylation and acetylation scores in different anatomical tumour sites. (A) Kaplan–Meier plots of overall survival corresponding to different anatomical sites of OSCC. (B) Kaplan–Meier plots of overall survival in two pooled groups of anatomical sites. The group “Tongue-FOM” contains tumour samples of tongue, floor of the mouth and oral cavity. The other anatomical sites are included in the group “Other sites”. (C) Distribution of samples in pooled anatomical sites according to combined H3K27 percentage (per) and perXin scores in OSCC patients. (D) Kaplan–Meier plots showing overall survival for different combined H3K27 percentage (per) and perXin scores within the groups of pooled anatomical sites. The significance symbols of p-values are the following: * for p-value < 0.05 and ** for p-value < 0.01. [file bmed-13-01-022s6.tif]
